# Investigation of digital and conventional methods for verifying the fitness of CAD/CAM crowns on abutments with different shapes

**DOI:** 10.1186/s40729-025-00632-8

**Published:** 2025-07-01

**Authors:** Michi Abe, Kazuhiro Kon, Shota Fukazawa, Hisatomo Kondo

**Affiliations:** 1 Division of Fixed Prosthodontics and Oral Implantology, Department of Prosthodontics, School of Dentistry, Iwate Medical University, 19-1 Uchimaru, Morioka, Iwate 020-8505 Japan; 2 Department of Fixed Prosthodontics and Oral Implantology, School of Dentistry, Aichi Gakuin University, Nagoya, Aichi Japan

**Keywords:** CAD/CAM technology, Digital scan, Dimensional accuracy, Intraoral scan

## Abstract

**Purpose:**

To evaluate the marginal and internal compatibility of computer-aided design and computer-aided manufacturing crowns produced via a digital workflow using an intraoral scanner, and to compare this digital-detection technique with the conventional fit test using silicone rubber (silicone-compatibility technique) on various abutments.

**Methods:**

Implant bodies were placed in the maxillary right central incisor and mandibular right first molar of reference models. Digital scans were acquired using an intraoral scanner, and abutments were prepared. Twenty-four crowns with a cement space of 70 μm were fabricated from the digital file of the abutment. The crown’s inner surface, abutment, and occlusal surface were scanned. The gaps between the crown and abutment were measured using stereoscopic image analysis software based on standard triangulated language data, and the accuracy of the fit was verified using silicone rubber.

**Results:**

Significant differences (*P* < 0.05) were observed between the silicone-compatibility and digital-detection techniques for the maxillary central incisor at the incisal edge and the palatal lower region, and for the mandibular first molar at the occlusal surface and the center of lingual axis. The digital-detection technique yielded values closer to 70 μm for the cement space. The values measured using the silicone-compatibility technique exhibited greater variation than those measured using the digital-detection technique.

**Conclusions:**

The novel digital-detection technique had superior or equivalent performance compared to the silicone-compatibility technique and could be beneficial for verifying crown fitness accuracy.

## Background

The clinical application of digital scans using intraoral scanners has grown dramatically owing to the spread of digital treatment [1]. The digital scan method is a technique wherein the details of the intraoral cavity and tooth surface shape are captured via direct photography. This method has been used to record the precise shapes of the abutment and contralateral tooth, and the occlusion as imaged data in real time. With the establishment of digital workflows, nonmetallic crowns fabricated using computer-aided design (CAD)/computer-aided manufacturing (CAM) technology have been increasingly applied in recent years. CAD/CAM technology is used to fabricate prostheses, such as crowns, inlays, and fixed partial dentures, for the restorative treatment of natural teeth [2–4], as well as for preoperative examination and prosthetic devices for oral implant treatment [5, 6]. Various manufacturers supply ceramic and resin blocks for crown fabrication using CAD/CAM technology, and the number of restorations has been increasing. Therefore, CAD/CAM technology is expected to simplify the fabrication method and improve the fit of prosthetic devices.

Several studies have described the fit of prostheses fabricated using digital scans obtained from intraoral scanners [7–12] and reported an improvement in accuracy. A major problem in creating prostheses using conventional methods is the errors that occur during the fabrication of the working model, such as shrinkage or expansion of materials because of the use of impression materials, plaster, wax, embedding materials, or metals. In contrast, the digital scan method, which uses an intraoral scanner, can generate impressions without the influence of distortions in impression materials, resulting in a reduced failure rate. However, a reliable and novel method for verifying the accuracy of the fit of prosthetic devices fabricated using CAD/CAM technology has not yet been established, and most reports have used the conventional method with silicone rubber [13, 14]. A prosthesis with poor accuracy can lead to mismatches, which can result in serious complications, such as prosthesis screw loosening and fracture of the implant protheses. Moreover, it can have a significant impact on the peri-implant soft tissue inflammation by plaque accumulation resulting from mismatches.

Several studies have evaluated the accuracy of intraoral scanners [15–18], which have been increasingly applied in oral implant treatment in recent years [19–21]. Recent studies have reported that the scanner yields similar or better accuracy than the conventional method using silicone rubber impression materials [9, 22]. However, few studies have evaluated the conformity of the inner surface of the prosthesis and abutment teeth bonded together in the same way as in clinical practice.

This study aimed to evaluate the conformity of the inner surface of crowns fabricated using a digital workflow and to compare the conventional fit test using silicone rubber (silicone-compatibility technique) with a novel inspection method using an intraoral scanner (digital-detection technique) on various abutments. The null hypothesis was that the shape of the abutment tooth did not affect the marginal fit and internal adaptation of the crown and the examination methods, a digital workflow and conventional silicone rubber fit test, had different detective ability.

## Methods

### Study design and setting

This study was designed as a laboratory-based comparative study for fitness evaluation of CAD/CAM-fabricated crowns on two types of abutments by using digital and physical methods. The digital method used an intraoral scanner, and the physical method used pressure-indicating silicon material. The abutments were fabricated with a 0.5-mm or 1-mm rounded shoulder finish.

### Manufacture of the reference model

**Fig. 1:**
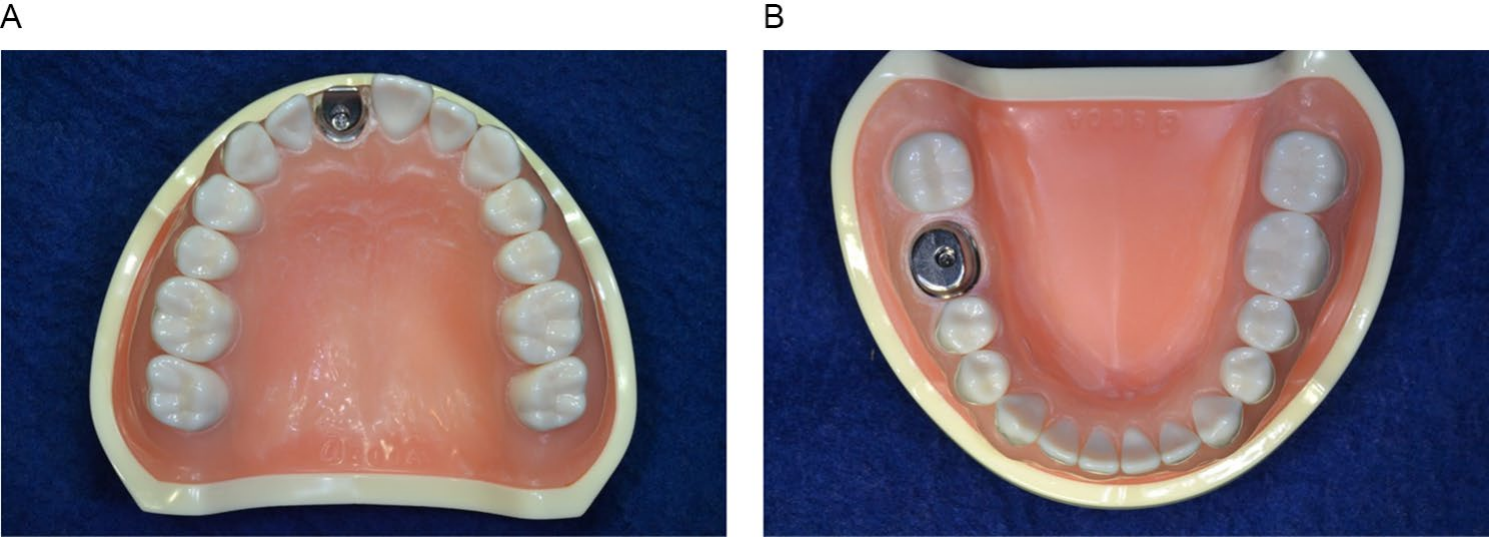
Reference model. **A**, Maxillary right central incisor. **B**, Mandibular right first molar

The two dental implants (maxillary right central incisor: Roxolid^R^ Bone Level, 3.3 × 10.0 mm NC; mandibular right first molar: Roxolid^R^ Bone Level, 4.1 × 10.0 mm RC; Straumann, Basel, Switzerland) were placed in the maxillary right central incisor region of the maxillary model and mandibular right first molar region of the mandibular model (D18FE-500 A-QF, NISSIN Dental Products, Kyoto, Japan) using an immediate polymerization resin(UNIFAST Trad, GC, Tokyo, Japan), respectively (Fig. 1).

### Abutment fabrication

**Fig. 2:**
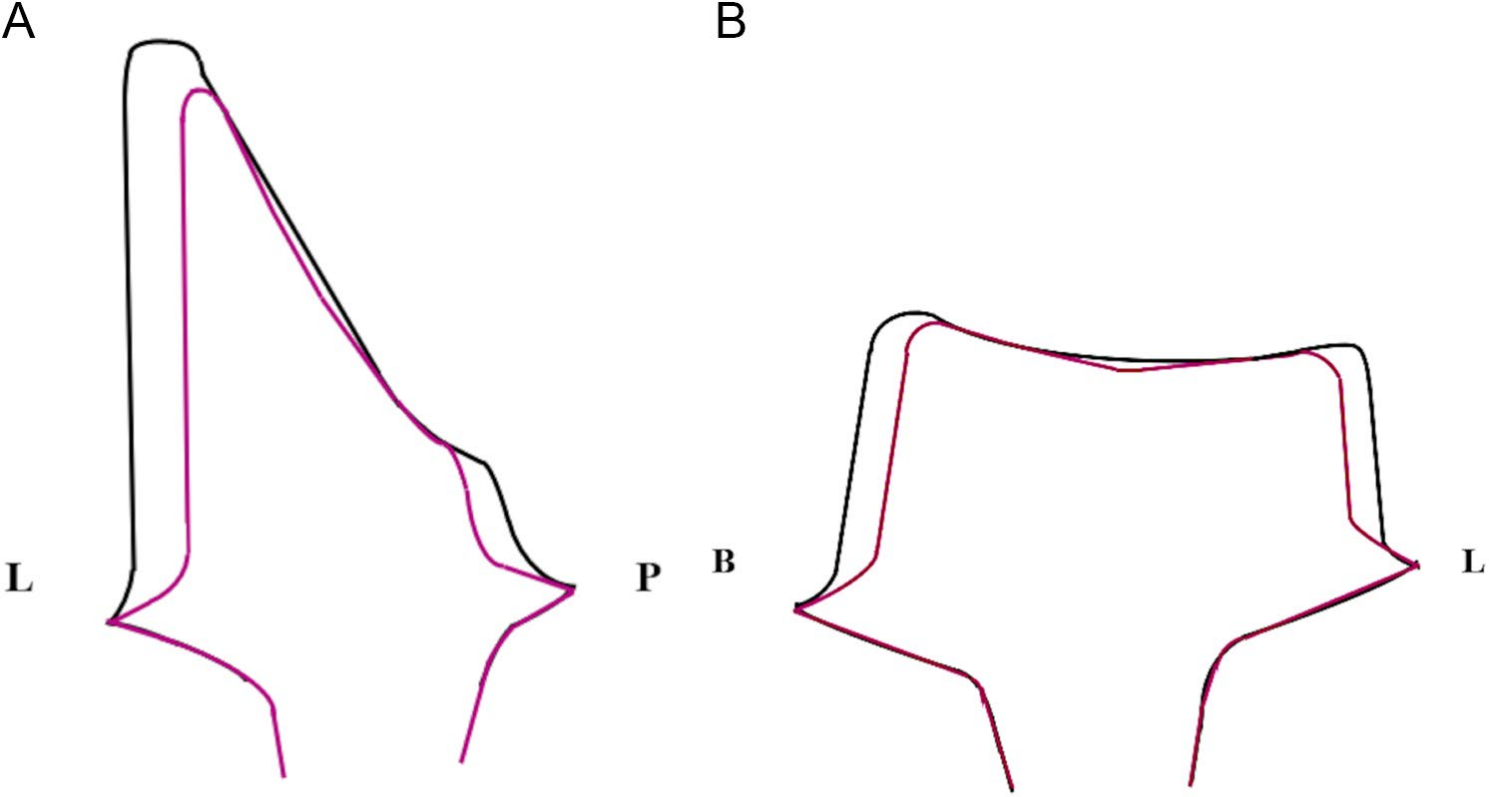
**A**, Titanium abutment in the maxillary right central incisor. **B**, Titanium abutment in the mandibular right first molar. Black line: a total convergence of 6 degrees, and a rounded shoulder finish line of 0.5- mm with rounded line angles. Red line: a total convergence of 7 degrees, and a rounded shoulder finish line of 1- mm with rounded line angles

The scan body (iOFLO, Dentsply Sirona, Bensheim, Germany) was attached to the body of the implant by hand tightening, and a digital scan was performed using an intraoral scanner (CEREC Primescan, Dentsply Sirona). Custom CAD/CAM made titanium abutments (Atlantis, Dentsply Sirona) were fabricated in 2 patterns in the maxillary right central incisor (maxillary right central incisor/0.5 mm: a total convergence of 6 degrees, a rounded shoulder finish line of 0.5 mm with rounded line angles; maxillary right central incisor/1 mm: a total convergence of 7 degrees, a rounded shoulder finish line of 1 mm with rounded line angles) (Fig. 2A) and 2 patterns in the mandibular right first molar (mandibular right first molar/0.5 mm: a total convergence of 6 degrees, a rounded shoulder finish line of 0.5 mm with rounded line angles; mandibular right first molar/1 mm: a total convergence of 6 degrees, a rounded shoulder finish line of 1 mm with rounded line angles) (Fig. 2B). Six abutments were used at each site.

### Crown fabrication

**Fig. 3:**
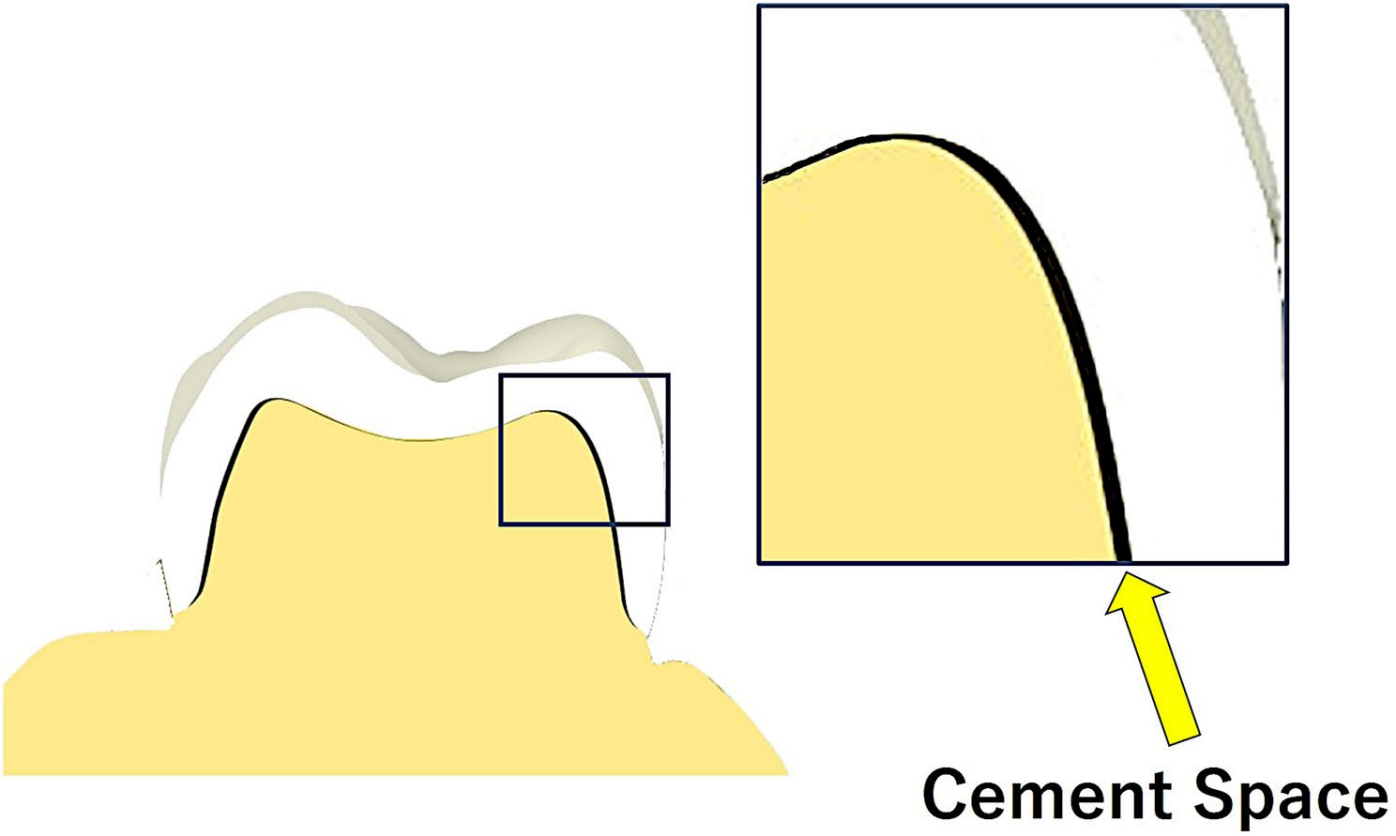
Schematic drawing of Cement Space. CAD/CAM crown was fabricated by 70 μm cement space setting

The crowns were designed using a CAD/CAM system (CEREC Primescan, Dentsply Sirona) with digital files of the abutments (core files) and fabricated using a milling machine (CEREC MCX, Dentsply Sirona). A hybrid resin composite block (Gamma Theta, YAMAKIN, Kochi, Japan) was used as the crown material. The milling bar was replaced after the fabrication of the five crowns, and the cement space was set to 70 μm (Fig. 3). The milling machine was configured to a normal mode that considers undercuts in the abutment; in total, 24 crowns were produced. Six crowns were fabricated for each abutment.

### Silicone-compatibility technique

**Fig. 4:**
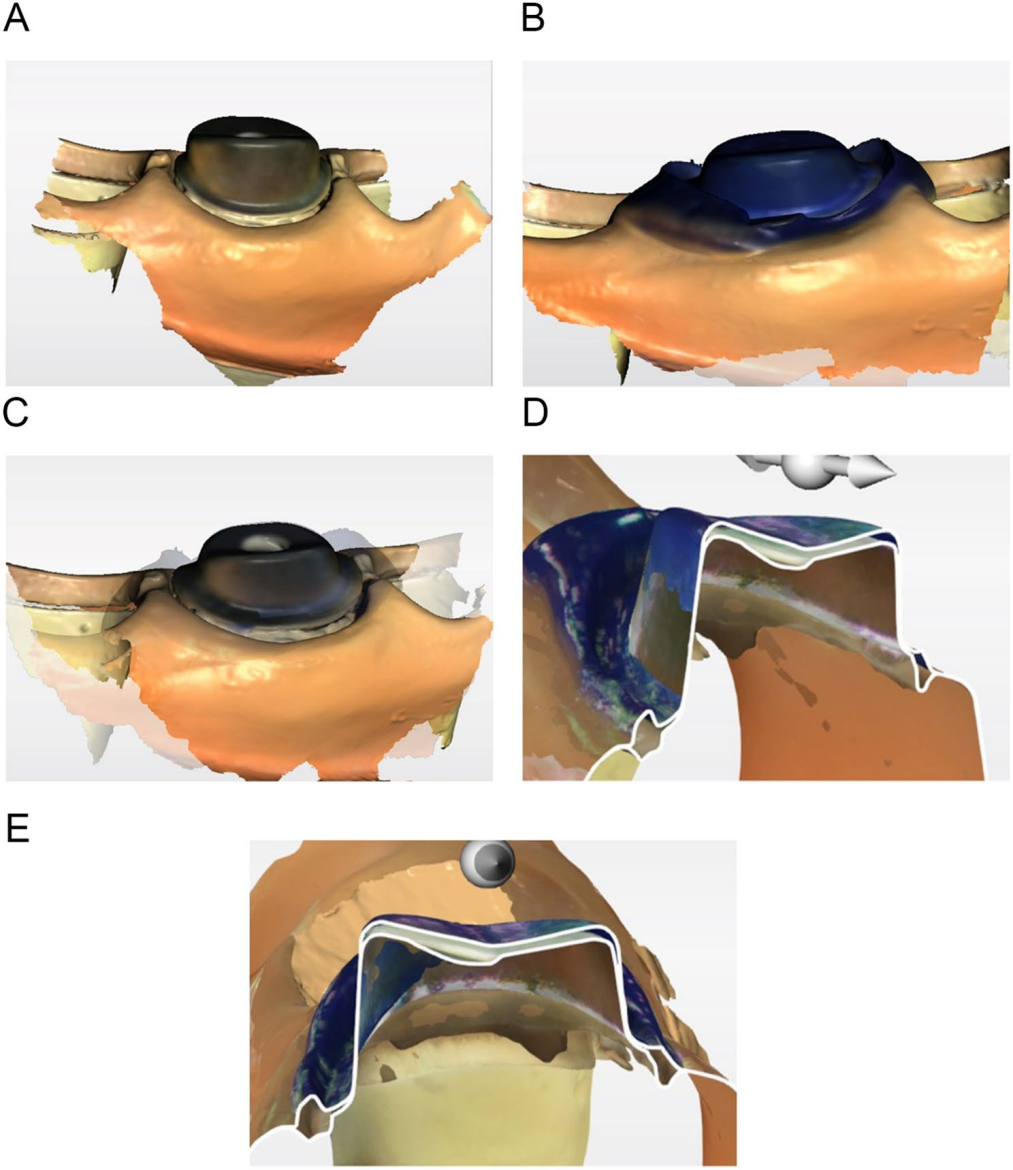
Silicone-compatibility technique. **A**, Optical impressions of the abutment. **B**, Optical impressions of the pressure-indicating silicone material remaining on the abutment. **C**, Superimposed image of implant abutment and pressure-indicating silicone material. **D**, **E**, Slice sections. (*n* = 6 for each group)

The crowns with a thin layer of separating material (washable SEP, SUN MEDICAL, Shiga, Japan) applied to the inside were filled with pressure-indicating silicone material (BLUE SILICONE, GC, Tokyo, Japan), were sufficiently hand pressed onto an abutment, and were removed carefully after curing. The same person performed the experiment, and the technique was standardized. Optical impressions of pressure-indicating silicone material on the abutment and the abutment alone were obtained using an intraoral scanner for each abutment in the 0.5-mm (*n* = 6) and 1-mm (*n* = 6) groups. A total of 24 standard triangulated language (STL) data were obtained for each group (Fig. 4).

### Digital-detection technique

**Fig. 5:**
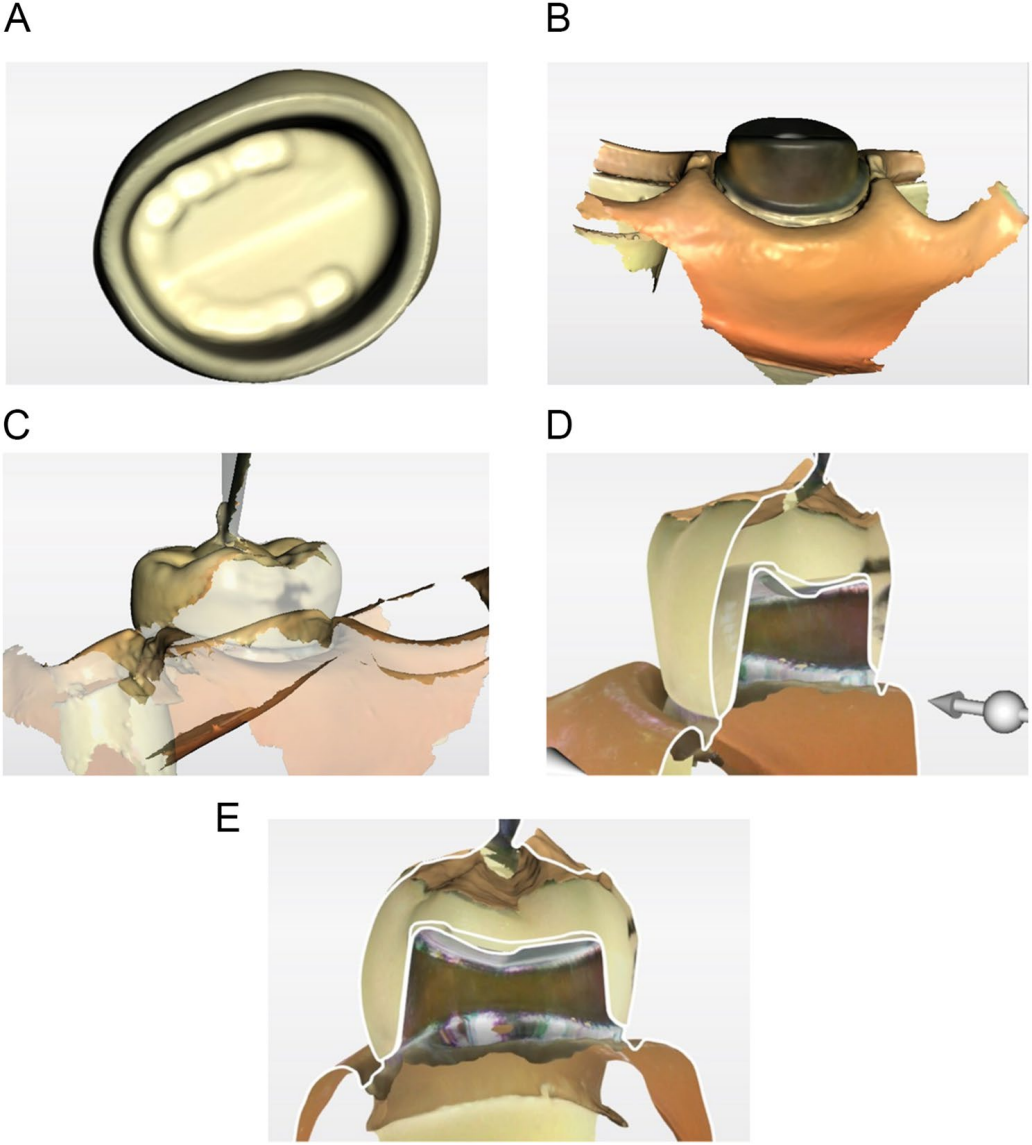
Digital-detection technique. **A**, Optical impressions of the crown inner surface and margin. **B**, Optical impressions of the abutment. **C**, Optical impressions of the crown attached to the abutment. **D**, **E**, Slice section. (*n* = 6 for each group)

Optical impressions of the crown inner surfaces, abutments alone, and crowns sufficiently hand pressed and attached to the abutments were obtained for each abutment in the 0.5-mm (*n* = 6) and the 1-mm (*n* = 6) groups. A total of 24 STL data were obtained for each group (Fig. 5).

### Measurement of the gap between the inner surface of the crown and the abutment

**Fig. 6:**
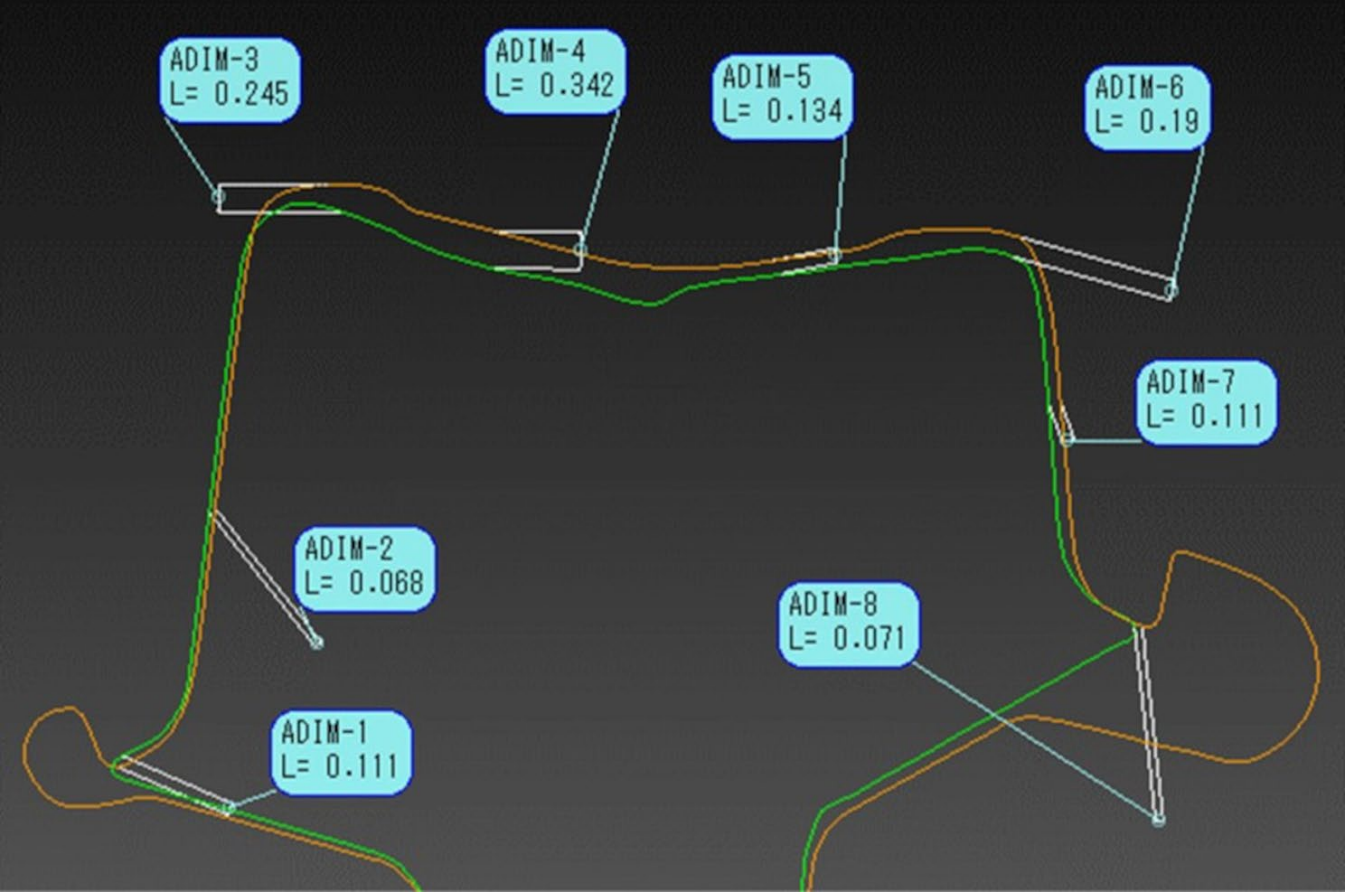
Stereoscopic image analysis using software. The STL data for the silicone-compatibility technique and digital-detection techniques were acquired and transferred to the software for stereoscopic image analysis to measure the gap between the crown and the abutment

The STL data for the silicone-compatibility technique and digital-detection techniques were acquired and transferred to a software for stereoscopic image analysis (spGauge 2019.1 (64-bit), Almonikos, Shizuoka, Japan). The best-fit algorithm was used to superimpose data cross-sectional lines were created from the point cloud data, and measure the gap between the crown and abutment (average ± SD) (Fig. 6).

**Fig. 7:**
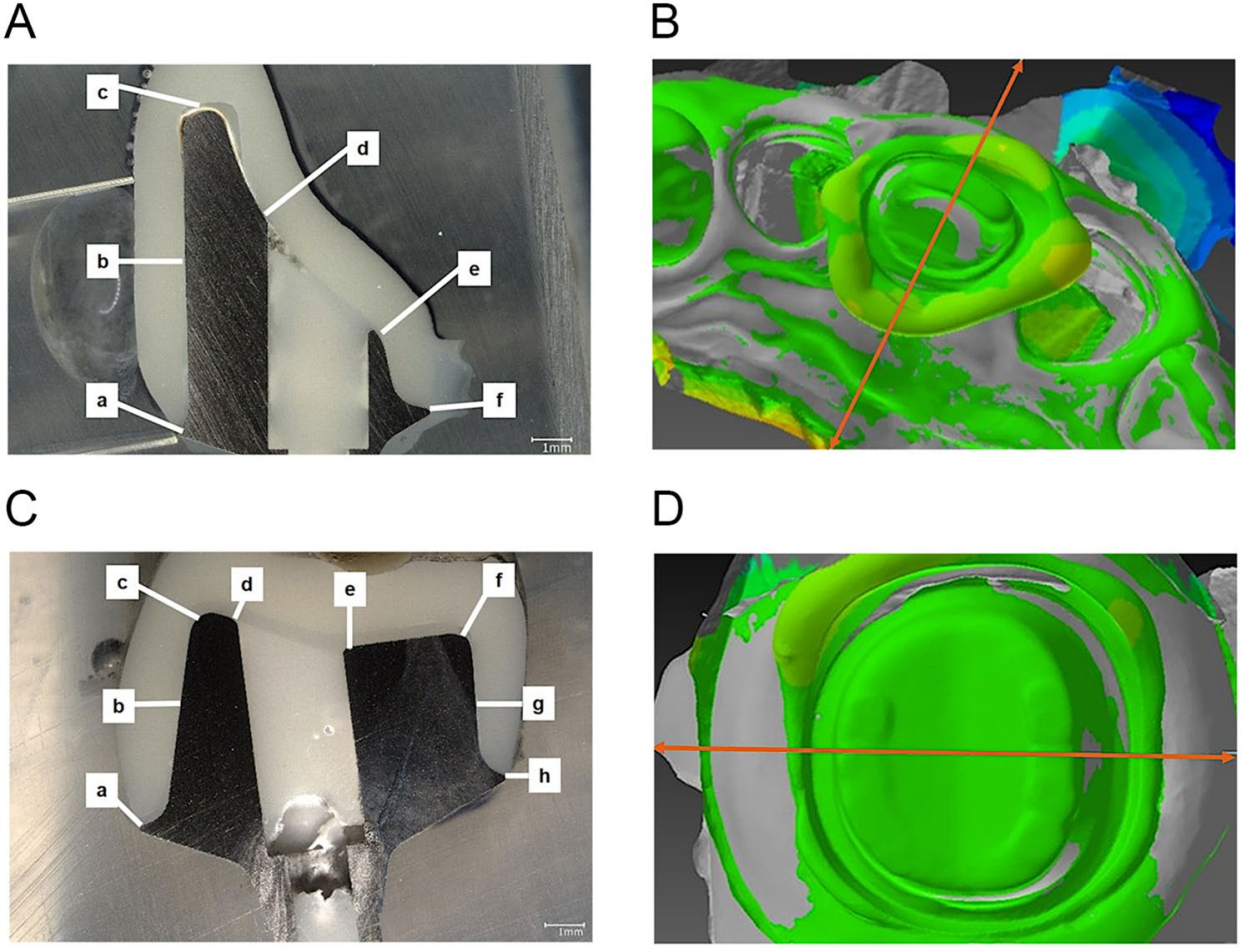
Measurement site. **A**, Maxillary right central incisor: the labial margin (**a**), the center of the labial tooth axis (**b**), the center of the incisal edge (**c**), the upper region of the palatal access hole (**d**), the lower region of the palatal access hole (**e**), and the palatal margin (**f**). **B**, Abutment cut (orange line) in the buccolingual direction. **C**, LR6: Buccal margin (**a**), center of the buccal tooth axis (**b**), corner of the buccal occlusal surface (**c**), buccal region of the access hole (**d**), lingual region of the access hole (**e**), corner of the lingual occlusal surface (**f**), center of the lingual tooth axis (**g**), and lingual margin (**h**). **D**, Abutment cut (orange line) in the buccolingual direction

The abutment was sectioned in the buccolingual direction and the following 6 sites were selected for the maxillary right central incisor abutment 0.5- mm and 1- mm: the labial margin (a), the center of the labial tooth axis (b), the center of the incisal edge (c), the upper region of the palatal access hole (d), the lower region of the palatal access hole (e), and the palatal margin (f). The following eight sites were selected for mandibular right first molar abutment 0.5- mm and 1- mm: the buccal margin (a), the center of the buccal tooth axis (b), the corner of the buccal occlusal surface (c), the buccal region of the access hole (d), the lingual region of the access hole (e), the corner of the lingual occlusal surface (f), the center of the lingual tooth axis (g), and the lingual margin (h) (Fig. 7).

### Statistical analysis

In total, 336 measurements were performed (4 groups of 6 specimens with 6 or 8 measurement locations per specimen). All data were analyzed by statistical software (R4.3.1, R Foundation, Wien, Austria). Normality and equal of variance of the experimental data were tested by the Shapiro-wilk and Bartlett tests, respectively. One-way analysis of variance (ANOVA) was performed for those where normality and equal of variance were confirmed, and the post-hoc test was Tukey’s honest significant difference (HSD). For those that did not meet the above criteria, the Kruskal-Wallis test was performed as a nonparametric test and the Dunn. test as a post hoc.

A Comparison of abutment morphology (Fig. 10), Variance was tested using the F-test. If the variances were equal, Paired t test was performed, otherwise if the variances were not equal, the Welch’s t test was performed. P value < 0.05 was considered to indicate statistical significance.

**Fig. 8:**
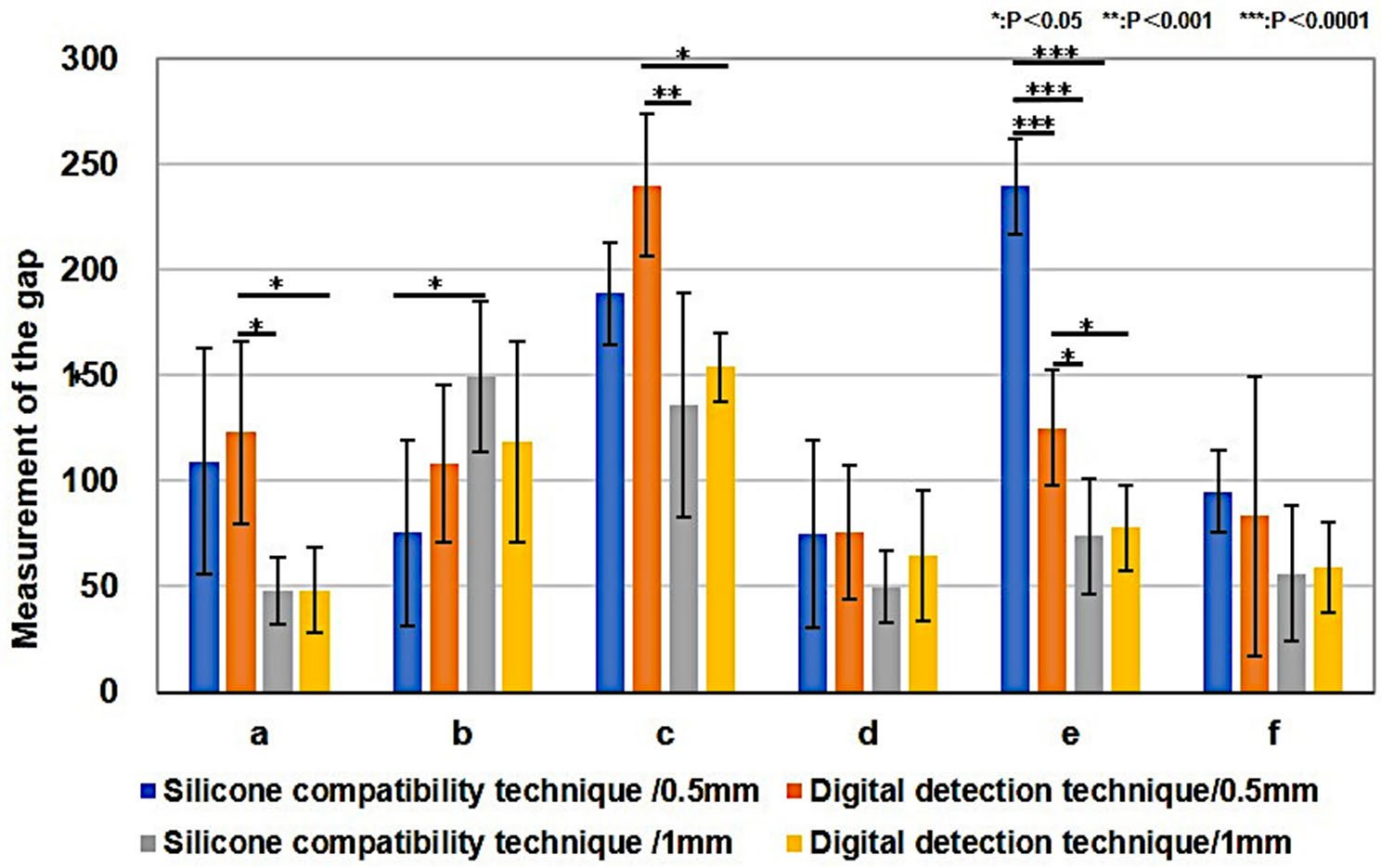
Maxillary Right Central Incisor Measurement Results. Blue bar graph; silicone compatibility technique/ total convergence of 6 degrees, a rounded shoulder finish line of 0.5 mm, Orange bar graph; digital detection technique/ total convergence of 6 degrees, a rounded shoulder finish line of 0.5 mm, Silver bar graph; silicone compatibility technique/ total convergence of 7 degrees, a rounded shoulder finish line of 1 mm, Yellow bar graph; digital detection technique/ total convergence of 7 degrees, a rounded shoulder finish line of 1 mm. Significant differences (*P* < 0.05) were found between the values of silicone-compatibility and the digital-detection techniques at measurement sites c (center of the incisal edge: *P* = 0.0006/0.5 mm and 1 mm) and e (lower region of the palatal access hole: *P* = 0.0000/0.5 mm). **b**, **c**, **d**, **e** by ANOVA, **a**, f by Kruskal-Wallis test

## Results

**Fig. 9:**
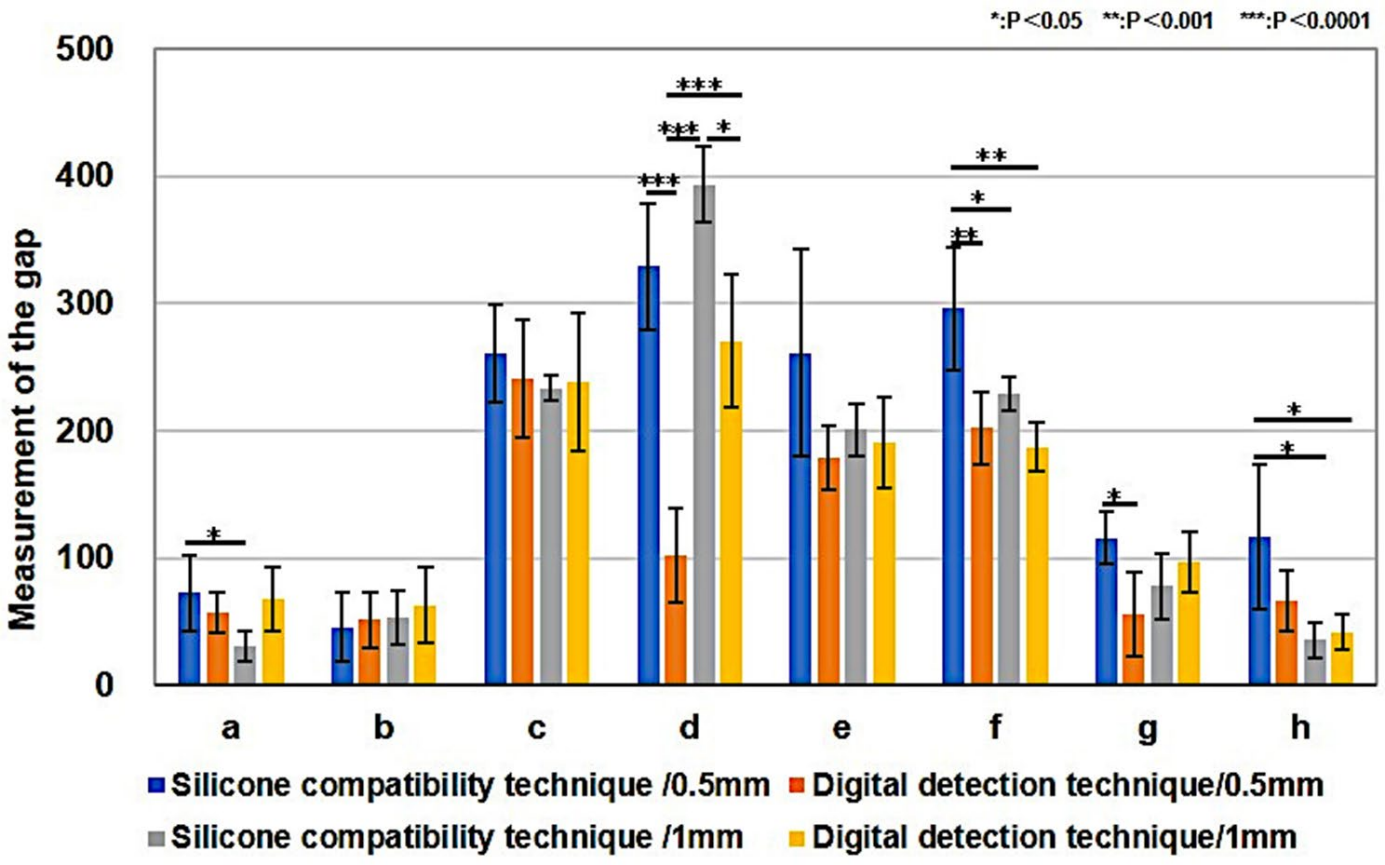
Mandibular Right First Molar Measurement Results. Blue bar graph; silicone compatibility technique/ total convergence of 6 degrees, a rounded shoulder finish line of 0.5 mm, Orange bar graph; digital detection technique/ total convergence of 6 degrees, a rounded shoulder finish line of 0.5 mm, Silver bar graph; silicone compatibility technique/ total convergence of 6 degrees, a rounded shoulder finish line of 1 mm, Yellow bar graph; digital detection technique/ total convergence of 6 degrees, a rounded shoulder finish line of 1 mm. Significant differences (*P* < 0.05) were found between values of the silicone-compatibility and digital-detection techniques at measurement sites d (the buccal region of the access hole: *P* = 0.0000/0.5 mm, *P* = 0.0010/1 mm) and g (the center of the lingual tooth axis: *P* = 0.0085/0.5 mm). **a**, **b**, **d**, **g** by ANOVA, **c**, **e**, **f**, **h** by Kruskal-Wallis test. The values obtained using the digital-detection technique were closer to the set cement spacing of 70 μm

The silicone-compatibility and digital-detection techniques at each measurement point was measured. In maxillary right central incisors, significant differences were found between values of silicone-compatibility and digital-detection techniques at measurement sites c (center of the incisal edge: *P* = 0.0006/0.5 mm and 1 mm) and e (lower region of the palatal access hole: *P* = 0.0000/0.5 mm) (Fig. 8). In mandibular right first molars, significant differences were found between values of the silicone-compatibility and digital-detection techniques at measurement sites d (the buccal region of the access hole: *P* = 0.0000/0.5 mm, *P* = 0.0010/1 mm) and g (the center of the lingual tooth axis: *P* = 0.0085/0.5 mm) (Fig. 9). In the maxillary right central incisor and mandibular right first molar, gap measurements were narrower in the axial region and wider on the occlusal surface and incisal edge. Furthermore, the silicone-compatibility technique revealed a greater variation in values than the digital-detection technique.

**Fig. 10:**
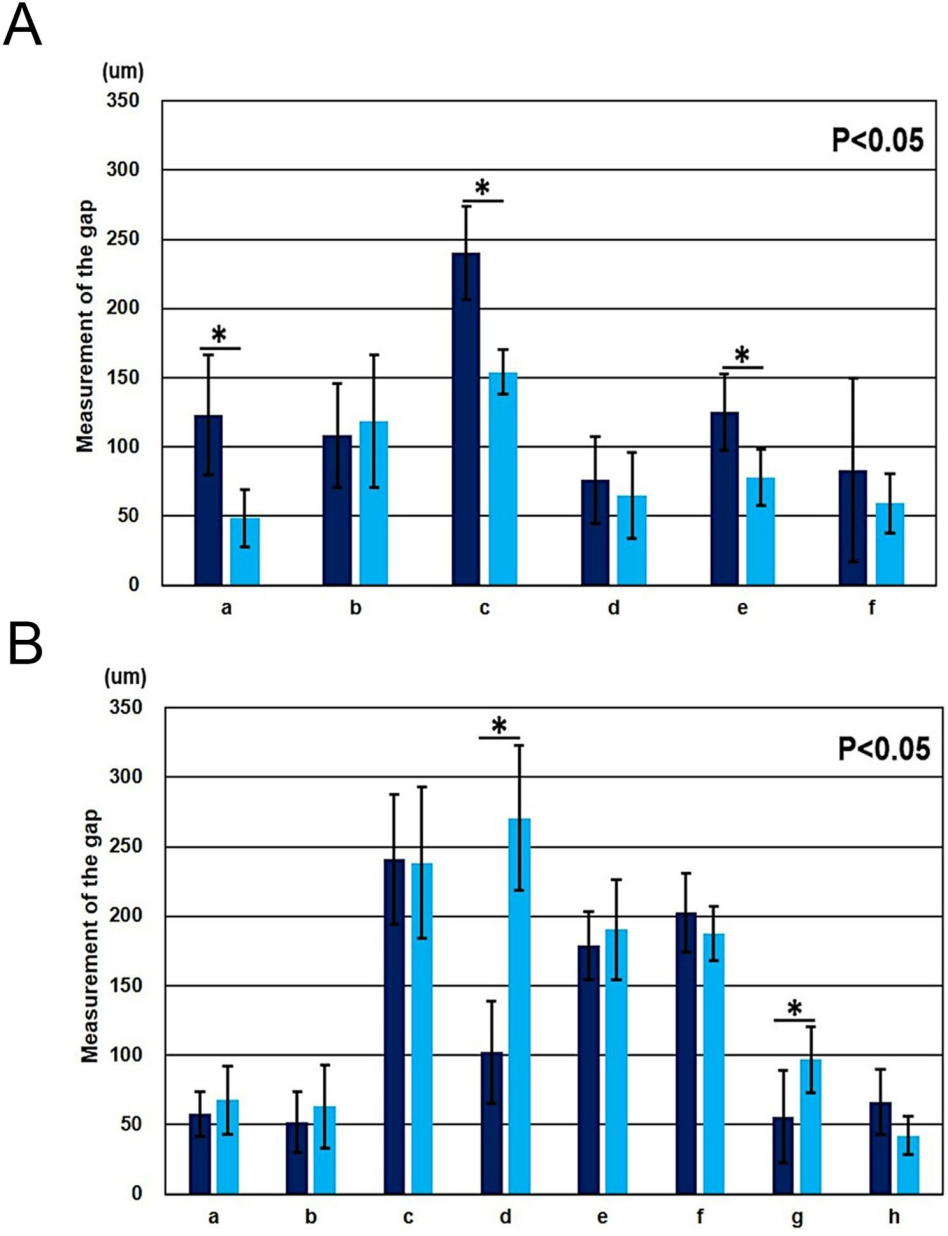
Comparison of abutment-tooth morphology by the digital-detection technique. A, Maxillary right central incisor. A significant difference (*P* < 0.05) was found between the values of the 0.5 mm and 1 mm abutment forms at measurement sites a (labial margin: *P* = 0.005678), c (center of the incisal edge: *P* = 0.000449), and e (lower region of the palatal access hole: *P* = 0.001134). a, b, c, d, e by Paired t test, f by Welch’s t test. B, mandibular right first molar. A significant difference (*P* < 0.05) was found between the values of the 0.5 mm and 1 mm abutment forms at measurement sites d (buccal region of the access hole: *P* = 0.00015) and g (center of the lingual tooth axis: *P* = 0.04856). **a**, **b**, **c**, **d**, **e**, **f**, **g** by Paired t test, **h** by Welch’s t test

Regarding abutment-tooth morphology, almost the same cement space was observed, except at a few sites (Fig. 10). Based on the aforementioned results, definite grooves were observed on the internal surfaces of the cusps in the CAD/CAM crowns, and these values were significantly larger than those in the set cement space.

## Discussion

Advances in digital workflows have enabled the fabrication of prostheses without the requirement for conventional silicone rubber impression making and modeling. An advantage of a digital workflow is that it allows objective data to be obtained and stored. The accuracy of digital scans obtained using intraoral scanners, which affect the fit of the crowns and abutments, has been reported to be equivalent to or more accurate than that of conventional methods using silicone rubber [14–16], and their accuracy has continued to improve. However, few studies have described methods to verify the fit of crowns fabricated via a digital workflow, and the accuracy of this fit is not well known.

Our study demonstrated that the space on the occlusal surface and at the incisal edge was significantly larger than the cement space, even when crowns were fabricated within 70 μm of the cement space. According to Reich et al., the internal fit discrepancy of single milled crowns was 80–92 μm for marginal gaps and 215–383 μm for occlusal spaces [23], which is similar to the results of this study. Therefore, our null hypothesis was rejected. The values in the axial region of the tooth were equal to or lower than those in the cement space, causing the crown’s inner surface to come into contact with the abutment and resulting in incompatibility [24]. Additionally, values at the margins were higher than those in the designed cement space, regardless of the measurement method used. Previous reports support the results of our study [25, 26]. These factors may be involved in tooth axis distortion during milling and consequent fractures at the margin of the crown.

The cement space is essential for the placement of prosthetic restorations on abutment teeth. However, no clear guidelines regarding the optimal amount of cement space have been established [27], and no method has been established to measure this space accurately [19–21]. Previous studies recommend cement spaces of at least 50–120 μm, and a smaller cement space reportedly results in a larger marginal discrepancy [28–30]. Therefore, the cement space was set to 70 μm in this study to verify the conformity of the inner surface.

The thickness of the prostheses plays an important role in the preparation of abutments for CAD/CAM crowns because the strength of the material and extent of abutment removal are greater than those for full cast crowns. The degree of reduction and total convergence of a natural tooth are determined based on the thickness of the pulp, tooth structure, and the required retentive force of the tooth. It is recommended to maintain a total convergence of 6–12 degrees and a finish line of 0.5–0.8 mm to achieve adequate retentive force [31, 32]. In this study, experiments were conducted using two patterns with different total convergence degrees (6–7 degrees) and a chamfer finish line. In the central incisors, a rounded shoulder finish line of 1 mm showed better margin fit. In the molar region, no significant difference was observed due to differences in the finish line. The same detection capability was observed for both the silicone-compatibility technique and digital-detection techniques. Based on the above results, it was considered that even when there are differences in the morphology of the abutment design, the silicone-compatibility technique and digital-detection techniques yield equivalent results. However, no significant differences were observed at any of the sites. This can be attributed to the reduction being within the appropriate range.

Several studies have reported the use of silicone rubber and stereomicroscopes to verify fit accuracy. However, the method using silicone rubber (the silicone replica technique) is associated with complications, such as difficulty in crown removal and distortion and rupture of the silicone rubber [33]. In contrast, a digital-detection technique using three-dimensional image analysis software is a nondestructive and less invasive method that does not destroy the specimen and is easy to perform. In this technique, STL data were obtained from three digital scans of the crown, abutment, and crown attached to the abutment using this digital-detection technique, and the imaging data were superimposed to achieve the best fit to measure the gap volume from the three-dimensional image.

This study has some limitations. First, regarding standardization, the comparisons were not always accurate and may have been more reliable if the measurements had been performed at exactly the same sites for each method. However, this was extremely difficult. Second, this in vitro study included the use of a single scanning and milling system. The size of the milling bar and the fabrication conditions make it difficult to fabricate fine parts of the crown; it is necessary to consider how to adjust the milling machine, and performing reliable measurements is challenging. Third, the impact of software on the CAD/CAM process must be considered. However, no significant differences were observed in measurements, regardless of the attachment or evaluation method. Fourth, because there were specific points at which a > 100 μm gap was observed, it remains unclear whether our experimental condition could sufficiently reflect the actual clinical conditions. These issues should be resolved in future studies.

## Conclusions

The digital-detection technique proposed herein is a novel method for verifying the accuracy of crown fit that does not require a conventional cutting process and has superior performance in several aspects compared to the conventional silicone method. Therefore, the proposed method could be useful for verifying the accuracy of the fit of the crown. The gap between the crown and abutment was greater than the designated value of the cement space, set at 70 μm during design, suggesting that the total convergence degrees of the abutment and cement space need to be considered.

## Data Availability

No datasets were generated or analysed during the current study.

## Abbreviations

ANOVA: Analysis of variance
CAD: Computer-aided-design
CAM: Computer-aided-manufacturing
STL: Standard triangulated language
